# The Quality of Selected Essential Medicines Sold in Accredited Drug Dispensing Outlets and Pharmacies in Tanzania

**DOI:** 10.1371/journal.pone.0165785

**Published:** 2016-11-15

**Authors:** Eliangiringa Kaale, Vicky Manyanga, Mhina Chambuso, Jafary Liana, Edmund Rutta, Martha Embrey, Thomas Layloff, Keith Johnson

**Affiliations:** 1 School of Pharmacy, Muhimbili University of Health and Allied Sciences, Dar es Salaam, Tanzania; 2 Pharmaceuticals & Health Technologies Group, Management Sciences for Health, Dar es Salaam, Tanzania; 3 Pharmaceuticals & Health Technologies Group, Management Sciences for Health, Arlington, VA, United States of America; Institute of medical research and medicinal plant studies, CAMEROON

## Abstract

**Introduction:**

The purpose of this study was to investigate the quality of a select group of medicines sold in accredited drug dispensing outlets (ADDOs) and pharmacies in different regions of Tanzania as part of an in-depth cross-sectional assessment of community access to medicines and community use of medicines.

**Methods:**

We collected 242 samples of amoxicillin trihydrate, artemether-lumefantrine (ALu), co-trimoxazole, ergometrine maleate, paracetamol, and quinine from selected ADDOs and pharmacies in Mbeya, Morogoro, Singida, and Tanga regions. The analysis included physical examination and testing with validated analytical techniques. Assays for eight of nine products were conducted using high-performance thin-layer chromatography (HPTLC). For ALu tablets, we used a two-tiered approach, where tier 1 was a semi-quantitative Global Pharma Health Fund-Minilab^®^ method and tier 2 was high-performance liquid chromatography (HPLC) as described in *The International Pharmacopoeia’s* monograph for artemether-lumefantrine.

**Results and Discussion:**

The physical examination of samples revealed no defects in the solid and oral liquid dosage forms, but unusual discoloration in an injectable solution, ergometrine maleate. For ALu, the results showed that of 38 samples, 31 (81.6%) passed tier 1 testing and 7 (18.4%) gave inconclusive drug content results. The inconclusive ALu samples were submitted for tier 2 testing and all met the quality standards. The pass rate using the HPTLC and TLC/HPLC assays was 93.8%; the failures were the ergometrine maleate samples purchased from both ADDOs and pharmacies. The disintegration testing of the solid dosage forms was conducted in accordance with US Pharmacopeia monographs. Only two samples of paracetamol, 1.2% of the solid dosage forms, failed to comply to standards. The study revealed a high overall rate of 92.6% of samples that met the quality standards. Although the overall failure rate was 7.4%, it is important to note that this was largely limited to one product and likely due to poor distribution and storage rather than poor manufacturing practices.

**Conclusions:**

Over 90% of the medicines sold in ADDOs and pharmacies met quality standards. Policy makers need to reconsider ergometrine maleate’s place on the list of medicines that ADDOs are allowed to dispense, by either substituting a more temperature-stable therapeutically equivalent product or requiring those sites to have refrigerators, which is not a feasible option for rural Tanzania.

## Introduction

Medicines are a cost-effective solution to many health problems, as long as they are available and affordable to patients in need, meet quality standards, and are properly used. Unfortunately, however, access to quality-assured medicines remains a challenge for many low- and middle-income countries [1]. An initiative that addresses access to essential medicines and pharmaceutical services is the accredited drug dispensing outlet (ADDO) program, which was scaled-up successfully in Tanzania and also adapted for Uganda and Liberia [2]. The program, launched in 2003, is a public-private partnership where the privately owned shops have to meet a set of government standards related to personnel training and premises infrastructure to achieve accreditation. In turn, accredited shops are allowed to sell a select number of prescription-only medicines, including select antimicrobials. The government of Tanzania has now rolled out the ADDO program to all mainland regions with more than 9,000 shops accredited and more than 19,000 dispensers trained [2]. In addition, ADDOs have played a role in a number of other public health initiatives, including Integrated Management of Childhood Illness, increasing access to artemisinin-based combination therapy and long-lasting insecticide-treated nets and tuberculosis case-finding [3,4].

Poor drug quality, including falsified medicines, is a critical problem in every country, but particularly in low-resource settings. A study of drug samples from seven countries in southeast Asia and 21 countries in sub-Saharan Africa reported up to 35% failing chemical analysis, 46% failing to comply with packaging requirements, and up to 36% classified as falsified [5]. Chemical instability caused by tropical conditions may have adverse effects on quality attributes such as color, disintegration time, hardness, and dissolution rate [6]; in addition, the chemical instability of active ingredients in formulated pharmaceutical products has been linked to loss of biological action, and in some instances, with potential toxicities [7–12]. Studies have looked at the influence of tropical conditions on the stability of drugs and concluded that drugs meant for distribution to areas with tropical climates must be stability tested under appropriate conditions [6,13–16].

In this paper, we discuss the physical and chemical characteristics of selected essential medicines sold at ADDOs and pharmacies in Tanzania.

## Methods

### Study areas

We targeted three districts in each of four regions (12 districts total): Morogoro (Kilombero, Kilosa, and Morogoro Urban districts), Tanga (Handeni, Muheza, and Tanga districts), Mbeya (Mbarali, Mbeya Urban, and Mbozi districts), and Singida (Iramba, Singida Rural, and Singida Urban districts). Samples were collected from 24 randomly selected ADDOs (two per district) and eight randomly selected pharmacies (representing eight districts).

### Sampling

We selected nine different drug products from the ADDO authorized medicines list to sample: amoxicillin trihydrate (capsules and syrups), co-trimoxazole (tablets and suspensions), paracetamol (tablets), quinine (tablets and injections), artemether-lumefantrine (ALu) (tablets), and ergometrine maleate (injections). The selection criteria included pharmacological classification, demand, cost, and thermal stability. All nine products were purchased from an ADDO or pharmacy (if product was available) in quantities corresponding to their therapeutic indications. In the event of multiple suppliers of a single product, the least expensive product was purchased. If the product was available both as a unit-of-use (e.g., blister pack) and in bulk containers (e.g., 500 or 1000 tablets or capsules per container) from which the dispensed medicine was counted out and dispensed in an envelope, whichever product was least expensive was purchased. If a shop did not have a particular product on the list, the data collector skipped the purchase of that product in that ADDO or pharmacy. The following number of sampling units were collected for each product: amoxicillin trihydrate (66), co-trimoxazole (58), paracetamol (23), quinine (42), artemether + lumefantrine (38), and ergometrine maleate (15).

### Sample collection and transport

We trained data collectors, who were auditing shops as part of a larger study, to collect the drug product samples. The collectors were asked to purchase the products as if they wanted them for personal or family use and not as part of a study. After purchasing the medicines, they went out of the sight of the outlet, put the drugs into the bags or containers we had provided, and labeled them with the appropriate code. Then the collectors filled in the sample collection form provided with details for each sample: sample code number, product name (brand/trade name or generic name as applicable), name of active ingredient or ingredients, dosage form, and strength per administration unit. The details are important to help differentiate one sample from another. We instructed the data collectors on how to package the samples to ensure that they reached the testing site without damage. The collectors filled the packaging container with cotton, foam, or other suitable material to protect the samples during transportation. All containers were sealed and appropriately labeled.

The sample amounts collected for each of the medicines ranged from a minimum of 20 to a maximum of 42 administration units for oral solids, four vials or ampoules for injectable products, and two bottles for syrups and oral suspensions.

### Sample testing

We subjected all samples of the nine products to physical inspection including labeling and manufacturing and expiry dates. For solid formulations, inspection included color, shape, texture, cracks, spots, etc.; for liquids, it included color, particulate matter, and clumping. Physical inspection was followed by the disintegration test for solid dosage forms, and one of two assay approaches. For eight of the products, we used high-performance thin-layer chromatography (HPTLC) assays. For ALu, we used the semi-quantitative Global Pharma Health Fund -Minilab^®^ TLC assay as a tier 1 test, because the HPTLC methodology for this product is not available [17]. If the initial TLC results for the ALu samples were inconclusive, we then applied a tier 2 test with a high-performance liquid chromatography (HPLC) assessment as described in *The International Pharmacopoeia* [18].

### Data management and analysis

We entered data from the field and from the lab testing into the Statistical Package for the Social Scientist (SPSS) Version 17. Data were cleaned and analyzed to generate results tables, Z-scores, and p-values.

## Results and Discussion

Of the 242 total samples, 202 (84%) were collected from ADDOs, while 40 (16%) were collected from pharmacies. In addition, 170 (70%) were solid dosage forms (i.e., tablets, capsules, dry granules); whereas, 72 (30%) were liquid dosage forms (syrups, suspensions, injections) (Table 1).

**Table 1:** Distribution of samples by dosage form description and facility type (N = 243).

Sample dosage form	Facility type		Total
	ADDO	Pharmacy	
Tablets	111	16	127
Injections	20	7	27
Syrups	6	2	8
Suspensions	25	7	32
Dry granules	5	3	8
Capsules	35	5	40
**Total**	**203**	**40**	**242**

### Physical inspection

**Fig 1:**
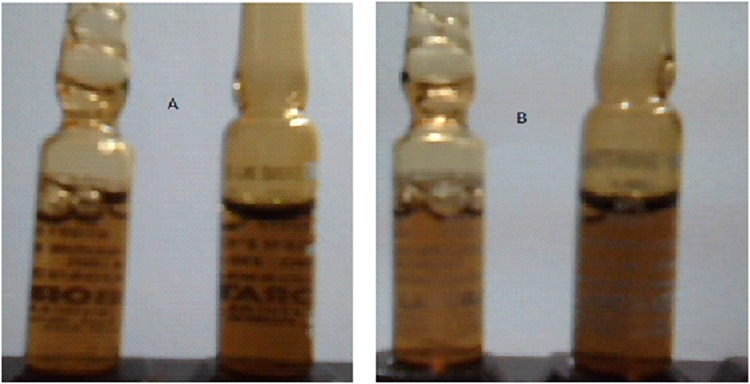
Photograph images of ergometrine maleate samples displaying color variations.

Almost all the 242 product samples (93.8%) we inspected had uniform shape and color, with no observed physical damage. However, 15 ergometrine maleate injections from both ADDOs and pharmacies were discolored. Multiple samples from the same outlet had the same level of discoloration, while samples taken from different outlets and regions showed large color differences, even if the manufacturers were the same. See Fig 1 for selected photo images.

### Minilab TLC assay and HPLC assay for ALu

The results of the Minilab TLC assays for ALu generally showed little difference across the four regions (Table 2). The tier 1 data indicated that out of 38 samples, 31 samples (81.6%) passed the test, and the results of the test for 7 samples (18.4%) were inconclusive. For the seven ALu samples with inconclusive results, we carried out tier 2 HPLC confirmatory testing using the *International Pharmacopoeia Monograph* for ALu [18]. All seven samples passed the confirmatory test.

**Table 2:** ALu tier 1 screening status by region (N = 38).

Region	Minilab^®^ outcome		Total
	Passed	Inconclusive	
Morogoro	8 (21.1%)	2 (5.3%)	10 (26.3%)
Tanga	5 (13.2%)	3 (7.9%)	8 (21.1%)
Mbeya	9 (23.7%)	1 (2.6%)	10 (26.3%)
Singida	9 (23.7%)	1 (2.6%)	10 (26.3%)
**Total**	**31 (81.6%)**	**7 (18.4%)**	**38 (100.0%)**

### Disintegration test

Of the 170 solid form samples, 161 were subjected to disintegration testing, where disintegration in less than 15 minutes indicates passing. Only 2 of 23 paracetamol tablet samples failed (8.7%), which was 1.2% of all solid dosage forms tested. This low failure rate generally suggests satisfactory formulation in the manufacturing process; however, local or regional pharmaceutical manufacturers, which is where Tanzania sources its paracetamol, may need to improve some practices, and regulatory authorities may need to increase monitoring of the quality of pharmaceutical products manufactured by local industries.

### HPTLC assay

Seven of the eight products assayed using HPTLC met the quality standards by complying with to 95 to 105% assay specifications. Only ergometrine maleate injections showed amounts of active pharmaceutical ingredient to be below the specified assay limits. The HPTLC assay pass rate was 93.4% with the failure rate of 6.6%—fully accounted for by ergometrine maleate injections (Table 3).

**Table 3:** Drug content determined by HPTLC or TLC/HPLC two-tier assay vs. compliance to quality standards (N = 243).

INN names of active ingredients	Drug content determination by HPTLC or TLC/ HPLC two-tier assay compliance to standards		Total
	Yes	No	
Amoxicillin trihydrate (tablets/capsules and syrups)	66 (27.3%)	0 (0.0%)	66 (27.3%)
Co-trimoxazole (tablets and oral suspensions)	58 (24.0%)	0 (0.0%)	58 (24.%)
Paracetamol (tablets)	23 (9.5%)	0 (0.0%)	23 (9.5%)
Quinine (injections and tablets)	42 (17.4%)	0 (0.0%)	42 (17.4%)
Artemether-lumefantrine (tablets)	38 (15.7%)	0 (0.0%)	38 (15.7%)
Ergometrine maleate (injections)	0 (0.0%)	15 (6.2%)	15 (6.2%)
**Total**	**227 (93.8%)**	**15 (6.2%)**	**242 (100.0%)**

We saw difference in assay values among ergometrine maleate samples, which mirrored the dramatic color differences in the samples (Table 4). Based on these observations, we hypothesize that the darker the sample, the lower the amount of ergometrine maleate active ingredient, which other studies have shown [14,16, 19]. Hogerzeil et al. established that injectable ergometrine maleate was very unstable under tropical conditions, particularly if stored unrefrigerated and exposed to light, where it may lose up to 20% of its potency per month [20]. Therefore, ergometrine maleate injections should be refrigerated and protected from light until given to the patient. Loss of potency can easily be detected by regular visual checks of the solution color. Any discoloration at all implies that the solution contains less than 90% of the stated amount of active ingredient and should not be used [20].

**Table 4:** Distribution of assay values of ergometrine maleate collected by region (N = 16).

	Average % active ingredient			
Region	Morogoro	Mbeya	Tanga	Singida
	43.41	65.97	51.71	69.65
	29.90	65.95	74.43	46.17
	15.56	62.63	31.83	-
	82.67	57.17	63.99	-
	-	85.90	-	-
	-	72.87	-	-
**Average % active ingredient**	**42.90**	**68.40**	**55.50**	**57.90**

Given that ADDOs are not required to keep refrigerators, the results were not surprising. However, it was surprising that all the ergometrine maleate samples from registered pharmacies, which *are* required to keep a refrigerator, also failed (Table 5). Given the instability of ergometrine maleate by heat and light, the results suggest that the failures in the pharmacy samples can be attributed to poor storage practices. Another possible cause could have been poor maintenance of ergometrine maleate’s cold chain requirement during shipment to the retail outlets.

**Table 5:** Ergometrine maleate assay results by facility type (N = 16).

Facility Type	Ergometrine assay		
	Pass	Fail	Total
ADDO	0 (0%)	12 (80%)	12 (80%)
Pharmacy	0 (0%)	3 (20%)	3 (20%)
**Total**	**0 (0%)**	**15 (100%)**	**15 (100%)**

### Overall evaluation of sample compliance with verification testing specifications

Table 6 summarizes the overall results of the samples’ compliance with verification testing specifications by facility type. This table pools together assessed parameters from visual inspection, disintegration testing, Minilab identification, and final assay. The study revealed a high overall passing rate of 92.6% for all samples tested against the quality standards, with an overall recorded failure rate of 7.4%, which is low compared to similar studies; for example, a review of 15 quality studies from mainly African and Asian countries had average failure rates up to 48% [21].

**Table 6:** Results of overall verification testing by facility type (N = 243).

	Facility Type		Total
	ADDO (n = 202 samples)	Pharmacy (n = 40 samples)	
Sample conformed with verification testing specification	188/225 (83.6%)	37/225 (16.4%)	225 (100.0%)
Sample did not conform with verification quality testing	15/17 (88.2%)	3/17 (17.7%)	17 (100.0%)
**Total**	**202/242 (83.5%)**	40/242 (16.5%)*	**242 (100.0%)**

* Not significant

In addition, to determine if there was a difference in the quality of medicines sold in registered pharmacies compared with ADDOs, we performed a Z-test of the two failure rate proportions. The comparison resulted in a p-value of 0.98404, which is not significant at p <0.05. Therefore, there is no evidence that the quality of drugs in the ADDO shops differed from those found in registered pharmacies. Similarly, a recently published study of the quality of antimalarials in Tanzania showed that ADDOs and unaccredited shops did not have more substandard samples than pharmacies did [15].

This was a risk-based assessment where we identified pharmaceutical quality issues with a higher risk profile [17, 22]. Unlike comprehensive quality assessments that include dissolution and impurity testing, we focused specifically on the identity and content of the medicines circulating in the retail pharmaceutical market in Tanzania to inform regulators and public health stakeholders of areas that need attention. As a result, limited available resources can be directed to achieve maximum impact. Based on our results, the public health problem with ergometrine maleate injections is evident. We believe the regulatory authority will launch a more holistic investigation on this and similar products to protect the public from being exposed to substandard products.

## Conclusions

This study revealed that the vast majority of medicines sold by ADDOs and pharmacies in Tanzania meet quality standards. Specifically, all sampled quinine injections and tablets, co-trimoxazole tablets and oral suspensions, and amoxicillin tablets, capsules, and syrups passed the disintegration tests, visual inspection, and HPTLC assays. Seven ALu samples gave inconclusive results with the Minilab TLC assay, but passed confirmatory HPLC testing. Two paracetamol samples failed disintegration testing, while all ergometrine maleate injection samples failed the active ingredient assay. This brings the overall failure rate to 7.4%

We expected the failure of ergometrine maleate injection samples because of their known sensitivity to tropical temperatures and light and given the fact that ADDOs do not have refrigeration. Policy makers need to consider substituting a more temperature-stable product for ergometrine maleate on the ADDO list of medicines, because refrigeration is not feasible in many of the remote areas where ADDOs are located. In addition, the failures seen from samples purchased from pharmacies suggest either lack of pharmacy adherence to good storage practices or poor cold chain control prior to product delivery to the retail outlets. We encourage the Tanzania Food and Drugs Authority to revise its postmarketing surveillance strategy to use ergometrine maleate as a surrogate marker for the integrity of the cold chain, which could increase the detection of problems with pharmaceuticals that are sensitive to heat and light. Finally, this study also demonstrates the feasibility of applying a risk-based model to test product quality that is independent of (or in addition to) ongoing post marketing surveillance conducted by the regulatory authority.
